# Nucleobase but not Sugar Fidelity is Maintained in the Sabin I RNA-Dependent RNA Polymerase

**DOI:** 10.3390/v7102894

**Published:** 2015-10-26

**Authors:** Xinran Liu, Derek M. Musser, Cheri A. Lee, Xiaorong Yang, Jamie J. Arnold, Craig E. Cameron, David D. Boehr

**Affiliations:** 1 Department of Chemistry, The Pennsylvania State University, University Park, PA 16802, USA; xil5090@psu.edu (X.L.); derekmusser43@gmail.com (D.M.M.); birxyang@scut.edu.cn (X.Y.); 2 Department of Biochemistry and Molecular Biology, The Pennsylvania State University, University Park, PA 16802, USA; cherilee1125@gmail.com (C.A.L.); jja5@psu.edu (J.J.A.); cec9@psu.edu (C.E.C.)

**Keywords:** enterovirus, poliovirus, Sabin, oral poliovirus vaccine, vaccine-associated paralytic poliovirus, vaccine-derived poliovirus, RNA-dependent RNA polymerase, polymerase fidelity

## Abstract

The Sabin I poliovirus live, attenuated vaccine strain encodes for four amino acid changes (*i.e.*, D53N, Y73H, K250E, and T362I) in the RNA-dependent RNA polymerase (RdRp). We have previously shown that the T362I substitution leads to a lower fidelity RdRp, and viruses encoding this variant are attenuated in a mouse model of poliovirus. Given these results, it was surprising that the nucleotide incorporation rate and nucleobase fidelity of the Sabin I RdRp is similar to that of wild-type enzyme, although the Sabin I RdRp is less selective against nucleotides with modified sugar groups. We suggest that the other Sabin amino acid changes (*i.e.*, D53N, Y73H, K250E) help to re-establish nucleotide incorporation rates and nucleotide discrimination near wild-type levels, which may be a requirement for the propagation of the virus and its efficacy as a vaccine strain. These results also suggest that the nucleobase fidelity of the Sabin I RdRp likely does not contribute to viral attenuation.

## 1. Introduction

Positive-strand RNA viruses are common causative agents of human disease, including the common cold, myocarditis, encephalitis, hepatitis, and paralytic poliomyelitis [1,2,3,4,5,6,7]. Poliovirus (PV) has been the subject of a largely-successful global eradication campaign [8,9]. These efforts have relied on the live attenuated oral poliovirus virus vaccine (OPV), and sustained in the developed world by inactivated poliovirus vaccine (IPV) [10]. Historically, OPV has been the favored treatment in the majority of countries, owing in part to its ease of use and lower cost [10]. However, most developed countries have transitioned to IPV because of OPV’s risk of vaccine-associated paralytic poliomyelitis (VAPP) and vaccine-derived polioviruses (VDPV) [11]. OPV is generally comprised of three vaccine strains empirically developed by Albert Sabin and colleagues [12,13,14]. The three Sabin vaccine strains all have mutations located in the virus’ internal ribosome entry site (IRES), which reduces the ability of PV to translate its RNA template within the neuronal cell [13,14]. Other mutations outside the IRES, including those within protein coding regions, may also contribute to viral attenuation [15,16,17,18,19,20]. The most abundant strain of the vaccine, Sabin I, has 57 mutations in its RNA sequence compared to the parental Mahoney strain, leading to 21 amino acid changes in viral proteins [21]. Four of these amino acid changes occur in the RNA-dependent RNA polymerase (RdRp) that is responsible for genome replication. We have previously shown that recombinant PV (*i.e.*, Mahoney background) encoding the Sabin-derived T362I mutation in the RdRp has a statistically significant reduction in viral virulence, likely because the T362I RdRp is a more error-prone polymerase than the “wild-type” (WT) enzyme [22]. These results are intriguing, and might suggest that the Sabin I RdRp also contributes to viral attenuation. Such findings would be intriguing in light of the suggestion that viruses encoding RdRp enzymes with altered fidelity might serve as live, attenuated vaccine candidates [23].

**Figure 1:**
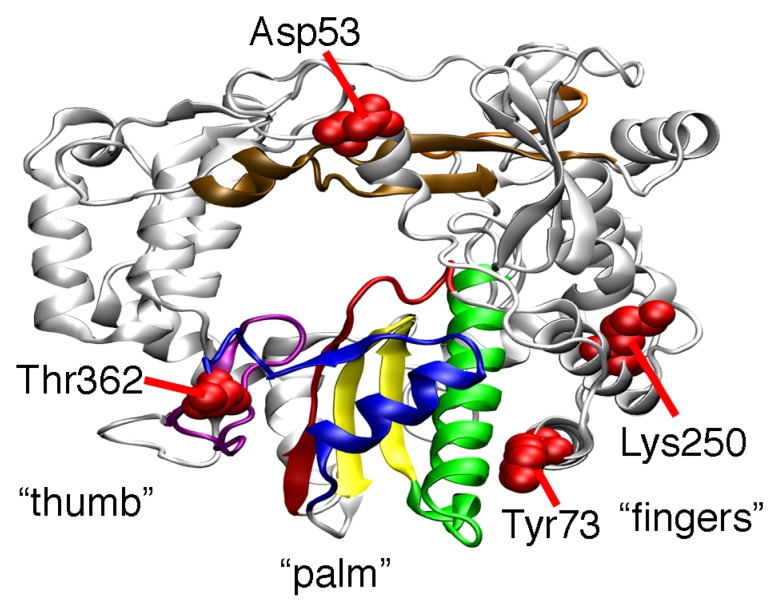
Locations of the amino acid substitutions in the Sabin I polymerase. The structure of the PV RdRp (PDB 1RA6 [33]) has fingers, palm, and thumb subdomains. Shown here is the “backside” of the protein, which allows easier visualization of the Sabin residues. The conserved structural motifs are colored (A, red; B, green; C, yellow; D, blue; E, purple; F, orange; G, brown). The locations of the amino acid residues changed in the Sabin I polymerase are indicated in red (*i.e.*, D53N, Y73H, K250E, and T362I).

PV RdRp has a highly-conserved canonical cupped right-hand structure with palm, thumb, and finger subdomains (Figure 1) [24]. There are seven conserved structural motifs, five of which (A to E) are located in the palm subdomain [25,26]. The T362I amino acid change occurs on structural motif D, which we have proposed is important in phosphodiester bond formation and nucleotide discrimination [27,28]. More specifically, motif D contains a highly-conserved lysine residue (Lys359 in PV RdRp), which we have proposed acts as a general acid to protonate the β-phosphate of the incoming nucleotide to facilitate bond breakage between the α- and β-phosphates and create a better pyrophosphate leaving group [28,29]. We have suggested that the active-site loop containing Lys359 fluctuates between “closed” and “open” conformations in which Lys359 is positioned and out-of-position for catalysis, respectively. Our previous nuclear magnetic resonance (NMR) studies are consistent with this proposal [22,29,30]. We have shown that the Sabin-derived T362I amino acid substitution alters the motions of motif D, allowing the enzyme to fluctuate more readily into a closed conformation even in the presence of incorrect nucleotide, leading to a less faithful polymerase [22].

Other amino acid substitutions in the Sabin I RdRp may also change RdRp function. The Y73H substitution has been shown to interfere with the initiation of RNA synthesis [19], which might help to explain why PV encoding the Y73H substitution is attenuated [17,20]. The D53N, Y73H, and T362I substitutions also contribute to the temperature sensitivity of the Sabin I vaccine [15,16,17,31]. The Sabin substitutions may also affect each other. Our previous molecular dynamics (MD) simulations suggest that the T362I amino acid substitution induces different nanosecond timescale motions in distant parts of the enzyme, including around Asp53 [22]. We have also previously noted anti-correlated motions between motif D and the α-helix containing Tyr73 [32]. It is also noted that the helices containing Tyr73 and Lys250 are packed closely together (Figure 1). Altogether, these results suggest that there are structural and dynamic connections between the various Sabin sites, which may contribute to the function of the Sabin I RdRp.

In this article, we show that although the T362I substitution by itself lowers RdRp fidelity, the Sabin I RdRp, encoding all four substitutions, discriminates against nucleotides with incorrect nucleobases at the same level as wild-type (WT) enzyme. These results may suggest that there was evolutionary pressure during the selection of the Sabin I virus to maintain an optimal level of RdRp fidelity. In contrast, the Sabin I RdRp is less faithful when selecting against 2′-modified nucleotides, which would not be under the same selection pressure.

## 2. Results

### 2.1. Sabin PV RdRp Discriminates against Incorrect Nucleobases, but not against Incorrect Sugars, to the Same Extent as WT RdRp

Our previous studies indicated that the T362I substitution in the PV RdRp lowers nucleotide fidelity, likely because it alters the structural dynamics of the motif-D active-site loop [22], an important structural component in nucleotide discrimination [29]. Recombinant PV encoding the T362I substitution was also attenuated in a mouse model of PV [22]. These results suggested that the Sabin I RdRp may also have altered fidelity and that its function may contribute to the attenuation of the Sabin I vaccine strain. One way to probe polymerase fidelity is to perform single nucleotide incorporation assays using purified enzyme [34,35]. As such, we produced a modified RdRp enzyme encoding all four amino acid substitutions (*i.e.*, D53N/Y73H/K250E/T362I). To ensure that the Sabin I RdRp was amenable to the single nucleotide incorporation assays, we first examined its ability to interact with RNA. We have previously shown that the T362I substitution does not significantly weaken interactions between enzyme and RNA compared to WT RdRp [22].

**Figure 2:**
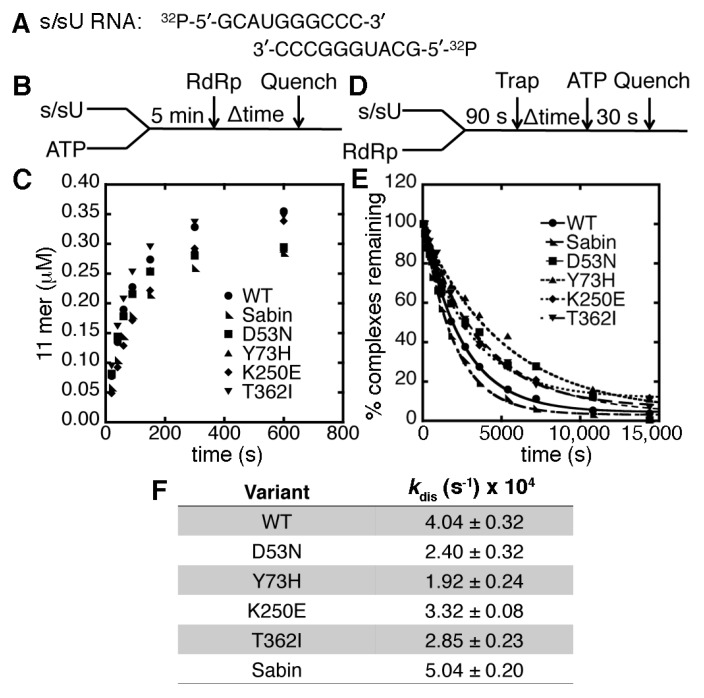
The Sabin amino acid substitutions do not substantially change the association or dissociation of RdRp complexes. (**A**) The s/sU RNA is ^32^P-labeled on the 5′ end for the association and dissociation assays; (**B**) the experimental design for the RdRp-RNA-NTP assembly assay. RNA (0.5 μM duplex) and 500 μM ATP (*i.e.*, which will template against U) were pre-incubated for 5 min at 30 °C before the addition of 1 μM RdRp. Reactions were quenched at the indicated times by adding 25 mM EDTA; (**C**) comparisons of the RdRp-RNA-NTP assembly assay for WT (●), D53N (■), Y73H (▲), K250E (♦), T362I (▼) and Sabin (◣) RdRp. The Sabin I RdRp contains all four amino acid substitutions (*i.e.*, D53N, Y73H, K250E and T362I); (**D**) the experimental design for the RdRp-RNA dissociation assay. RdRp (1 μM) and RNA (0.1 μM) were pre-incubated at 30 °C for 90 s before the addition of 100 μM unlabeled RNA (*i.e.*, “trap”). After the indicated times, the reaction buffer was mixed with 500 μM ATP and then quenched after 30 s by the addition of 25 mM EDTA; (**E**) the RdRp-RNA dissociation assays for WT (●), D53N (■), Y73H (▲), K250E (♦), T362I (▼) and Sabin (◣) RdRp. The lines represent the data fits to a single exponential function; (**F**) the RdRp-RNA dissociation rate constants derived from the data in panel (**E**). The dissociation rate constants for the RdRp variants are not substantially different from the WT enzyme.

The RNA template used in the kinetic assays was the symmetrical primer/template substrate (s/sU) that encodes for six complimentary base pairs and a four nucleotide overhang at the 5′ end (*i.e.*, 5′-GCAUGGGCCC-3′), which has been used in previous kinetic and NMR analyses of the PV RdRp [22,29,34,35,36]. The s/sU RNA has a uracil as the first templating base in the RNA duplex. The rate and yield of competent RdRp s/sU complexes for the Sabin I RdRp was highly similar to that of WT PV RdRp (Figure 2). The dissociation rate constants for the RdRp-RNA complexes were also very similar to that of WT PV RdRp (Figure 2). Experiments with single-substituted variants (*i.e.*, D53N, Y73H, and K250E) also did not reveal substantial differences from the WT results. Any small differences we observed between WT and variant RdRp enzymes were deemed not sufficient to interfere with the single nucleotide incorporation assays.

**Figure 3:**
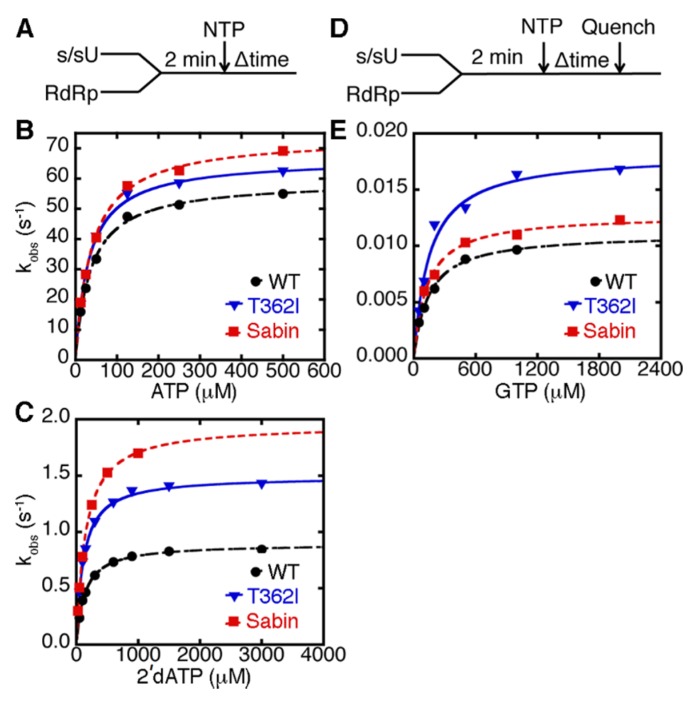
The Sabin I RdRp discriminates less against nucleotides with an incorrect 2′-deoxyribose sugar, but maintains nucleobase fidelity similar to WT enzyme (**A**) experimental design for the single nucleotide incorporation assay using ATP and 2′-dATP. RdRp (1 μM) was pre-incubated with s/sU RNA (1 μM), before being quickly mixed with equal volume of ATP or 2′-dATP with different concentrations. These reactions were monitored by fluorescence changes over time using a stopped-flow apparatus. Kinetic data for (**A**) AMP and (**B**) 2′-dAMP incorporation are plotted. The results for WT, T362I, and Sabin RdRp are shown in black, blue, and red, respectively. The lines represent data fit to a hyperbola function to give an apparent dissociation constant (*K*_d,app_) and a maximal rate constant for nucleotide incorporation (*k*_pol_); (**D**) the experimental design for the single nucleotide incorporation assay using GTP. RdRp (1 μM) was pre-incubated with s/sU RNA (1 μM) at room temperature for 3 min and then at the assay temperature of 30 °C for 2 min, before being quickly mixed with equal volume of GTP at different concentrations. In this case, RNA was ^32^P labeled on 5′-end; and (**E**) kinetic data for GMP incorporation are plotted in black, blue, and red for WT, T362I and Sabin RdRp respectively. The lines represent data fit to a hyperbola function to yield *K*_d,app_ and *k*_pol_ values.

We have previously investigated the nucleobase and sugar selectivity of the T362I variant using single nucleotide incorporation assays, which yield the maximal rate constant for nucleotide incorporation (*k*_pol_) and the apparent dissociation constant for the incoming nucleotide (*K*_d,app_) (Figure 3) [22,34,35]. These results indicated that the T362I variant had similar *k*_pol_ and *K*_d,app_ values for correct nucleotide incorporation as WT RdRp, but had higher catalytic efficiency for incorrect nucleotide incorporation, including those nucleotides with an incorrect sugar (*i.e.*, 2′-dNTP) or incorrect nucleobase [22]. We performed similar experiments on the Sabin I variant (Table 1). It should be noted that the correct nucleotide was ATP in this case, since it is templated against U, and so nucleotides with incorrect sugar and nucleobase were 2′-dATP and GTP, respectively.

The Sabin I RdRp had higher rates of single nucleotide incorporation than WT enzyme using ATP, 2′-dATP, and GTP (Table 1; Figure 3). The fidelity of nucleotide incorporation can be expressed according to the kinetic experiments as (*k*_pol_/*K*_d,app_)_correct_/(*k*_pol_/*K*_d,app_)_incorrect_, where (*k*_pol_/K_d,app_)_correct_ and (k_pol_/*K*_d,app_)_incorrect_ are the second-order rate constants for correct (*i.e.*, ATP) and incorrect nucleotide (*i.e.*, using 2′-dATP or GTP) incorporation respectively (Table 1). Our results suggested that the Sabin I RdRp had a similar ability to discriminate against nucleotides with incorrect nucleobase (*i.e.*, (*k*_pol_/*K*_d,app_)_ATP_/(*k*_pol_/*K*_d,app_)_GTP_) as WT enzyme (Table 1). However, Sabin I RdRp had a reduced ability to discriminate against nucleotides with a 2′-deoxyribose sugar (*i.e.*, (*k*_pol_/*K*_d,app_)_ATP_/(*k*_pol_/*K*_d,app_)_2’-dATP_) compared to WT enzyme. These results suggested that the other three amino acid substitutions in the Sabin I RdRp (*i.e.*, D53N, Y73H, K250E) may also impact RdRp fidelity.

### 2.2. The K250E Substitution is Unstable in Cell Culture

The nucleobase fidelity of the Sabin I RdRp was similar to that of WT enzyme (Table 1). Nonetheless, the Sabin amino acid substitutions may alter other functions of the RdRp to impact virus biology. To explore this idea, we attempted to encode recombinant PV (in Mahoney background) with the four substitutions occurring in the Sabin I polymerase. Unfortunately, this variant was not genetically stable and virus recovered from HeLa cells only retained the corresponding D53N/Y73H/T362I mutations. These results suggested that the K250E substitution was not stable in the Mahoney background, in the absence of other Sabin I mutations. Nonetheless, there was a possibility that the triple variant D53N/Y73H/T362I could be used in place of the Sabin variant for further biological characterization. Unfortunately for those studies, the D53N/Y73H/T362I variant had different sugar and nucleobase selectivities than the Sabin I variant (Table 1), and so we did not proceed with cell-based or mouse-based studies. There were also functional differences between the D53N/Y73H/T362I variant and T362I and WT RdRp enzymes (Table 1). Again, these results suggested that the other two substitutions, D53N and Y73H, had some effect on the rates and fidelity of nucleotide incorporation.

**Table 1:** The Sabin amino acid substitutions induce small changes in RdRp catalytic rates and fidelity.

Variant	NTP	*k*_pol_ (s^−1^)	*K*_d,app_ (μM)	*k*_pol_/*K*_d,app_ (μM^−1^·s^−1^)	*k*_pol,corr._/ *k*_pol,incorr._	(*k*_pol_/*K*_d,app_)_corr._/ (*k*_pol_/*K*_d,app_)_incorr._
WT	ATP	5.9 ± 0.1 × 10^1^	36 ± 2	1.6	–	–
D53N		6.2 ± 0.1 × 10^1^	39 ± 2	1.6	–	–
Y73H		4.6 ± 0.1 × 10^1^	40 ± 2	1.2	–	–
K250E		7.6± 0.1 × 10^1^	58 ± 3	1.3	–	–
T362I		6.7 ± 0.1 × 10^1^	33 ± 2	2.0	–	–
D53N/T362I		7.2 ± 0.1 × 10^1^	44 ± 2	1.6	–	–
D53N/Y73H/T362I		8.4 ± 0.2 × 10^1^	47 ± 4	1.8	–	–
Sabin		7.4 ± 0.1 × 10^1^	39 ± 2	1.9	–	–
WT	2′-dATP	8.9 ± 0.1 × 10^-1^	134 ± 4	6.7 × 10^−3^	70	240
D53N		9.3 ± 0.2 × 10^-1^	101 ± 9	9.3 × 10^−3^	70	170
Y73H		7.3 ± 0.1 × 10^-1^	117 ± 5	6.2 × 10^−3^	60	190
K250E		1.4 ± 0.0	174 ± 6	8.0 × 10^−3^	50	160
T362I		1.5 ± 0.0	112 ± 4	1.3 × 10^−2^	40	150
D53N/T362I		1.5 ± 0.0	132 ± 6	1.1 × 10^−2^	50	150
D53N/Y73H/T362I		1.4 ± 0.0	101 ± 9	1.4 × 10^−2^	60	130
Sabin		2.0 ± 0.0	145 ± 4	1.4 × 10^−2^	40	140
WT	GTP	1.1 ± 0.1 × 10^−2^	142 ± 15	7.7 × 10^−5^	5400	21,000
D53N		7.3 ± 0.8 × 10^−3^	91 ± 35	8.0 × 10^−5^	8400	20,000
Y73H		8.2 ± 0.5 × 10^−3^	154±31	5.3 × 10^−5^	5600	23,000
K250E		9.9 ± 0.8 × 10^−3^	160±41	6.1 × 10^−5^	7700	21,000
T362I		1.8 ± 0.1 × 10^−2^	149 ± 25	1.2 × 10^−4^	3700	17,000
D53N/T362I		1.2 ± 0.1 × 10^−2^	115 ± 21	1.0 × 10^−4^	6000	16,000
D53N/Y73H/T362I		1.1 ± 0.1 × 10^−2^	128±12	8.6 × 10^−5^	7600	21,000
Sabin		1.3 ± 0.1 × 10^−2^	127 ± 16	1.0 × 10^−4^	5700	19,000
WT	2′-C-methyl ATP	1.2 ± 0.0	160 ± 9	7.5 × 10^−3^	50	210
Sabin		1.9 ± 0.0	129 ± 5	1.5 × 10^−2^	40	130

### 2.3. D53N, Y73H and K250E PV RdRp Present Different Fidelities for Sugar and Nucleobase Selection

The functional differences between the Sabin I, D53N/Y73H/T362I, and T362I variants suggested that the D53N, Y73H, and K250E substitutions all affect RdRp function. As such, we determined kinetic values for the other single-substituted variants (Figure 1; Table 1). Although the changes induced by the single substitutions were relatively small, we note that small changes in RdRp fidelity can lead to biological effects (e.g., [37,38,39,40]). All three variants (*i.e.*, D53N, Y73H, and K250E) were a little less selective against nucleotides with incorrect sugars compared to WT RdRp. However, there were different effects for nucleobase selection. The nucleobase selectivities for the D53N, Y73H, and K250E variants were lower than, higher than, and similar to that of WT RdRp, respectively (Table 1).

### 2.4. Allosteric Effects among the Sabin Amino Acid Substitutions

**Figure 4:**
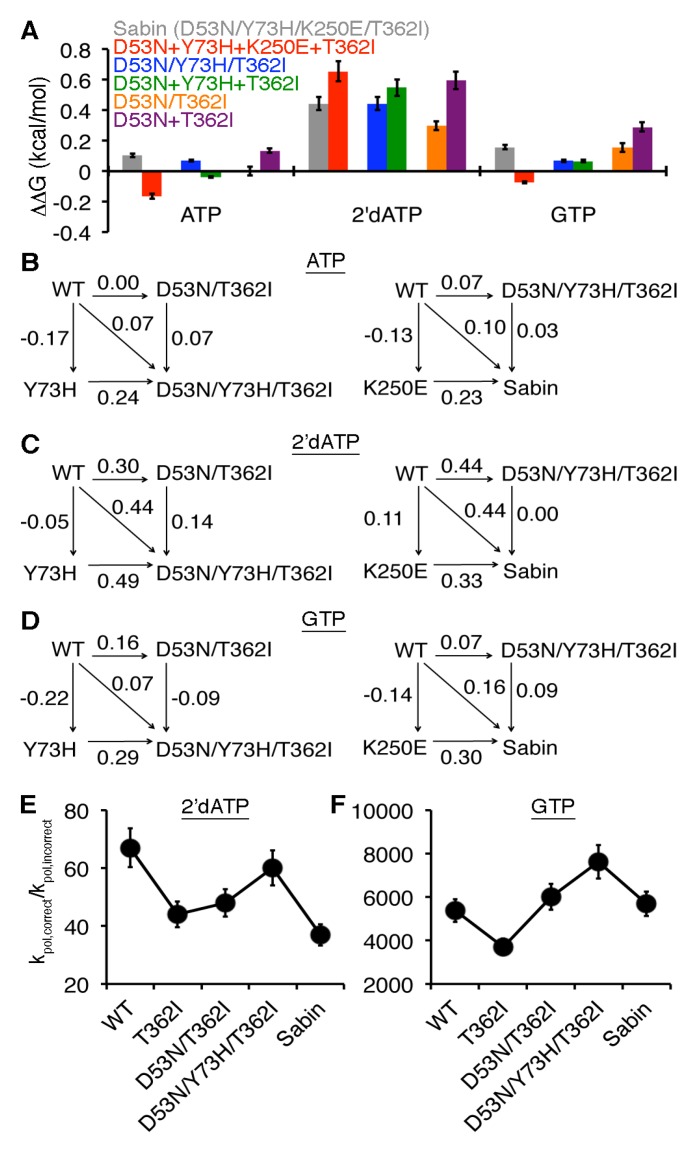
The Sabin I amino acid substitutions are cooperative. (**A**) Comparisons of single-substituted variants with multi-substituted variants. In this case, ΔΔG_X_ = RT ln((*k*_pol_/*K*_d,app_)_X_/(*k*_pol_/*K*_d,app_)_WT_) where X is a particular enzyme variant. The ΔΔG result for the Sabin variant with all four amino acid substitutions (D53N/Y73H/K250E/T362I) is in grey, and is compared to the sum of the effects for all four single variants in red (*i.e.*, ΔΔG(D53N) + ΔΔG(Y73H) + ΔΔG(K250E) + ΔΔG(T362I)). Likewise, the ΔΔG result for the triple variant D53N/Y73H/T362I is in blue and is compared to the sum of the effects for the three single variants in green. The ΔΔG result for the double variant D53N/T362I is in orange and is compared to the sum of the effects for the two single variants in purple. The ΔΔG values for the multi-variants are also compared using thermodynamics cycles when (**B**) ATP, (**C**) 2′-dATP, and (**D**) GTP are the incoming nucleotides (templated against U); all values are reported as kcal/mol. On the left, the ΔΔG values for the triple variant D53N/Y73H/T362I are compared to those of the double variant D53N/T362I and single variant Y73H. On the right, the ΔΔG values for Sabin variant with all four amino acid substitutions are compared to those of the triple variant D53N/Y73H/T362I and single variant K250E. (**E**) Sugar and (**F**) nucleobase selectivities are also compared between WT, T362I, D53N/T362I, D53N/Y73H/T362I, and the Sabin RdRp.

The changes induced by the four single-substituted variants do not readily explain the kinetic results with the Sabin I variant, suggesting that there may be allosteric interactions between the amino acid substitutions. Another way of characterizing protein variants is to analyze the thermodynamic effects of the amino acid substitutions. Similar to the methods of Fersht and Mildvan [41,42,43], we determined ΔΔG values where ΔΔG_X_ = RT ln((k_pol_/K_d,app_)_X_/(k_pol_/K_d,app_)_WT_) where X is a particular enzyme variant (N.B. in our analysis, variants that have a lower k_pol_/K_d,app_ value than WT enzyme will yield a negative ΔΔG value). In the case of the Sabin I variant, there were small thermodynamic effects for AMP and GMP incorporation, but more substantial effects for 2′-dAMP incorporation (Figure 4a). This type of analysis also allows us to determine if the four amino acid substitutions were additive (*i.e.*, ΔΔG_Sabin_ = ΔΔG_D53N_ + ΔΔG_Y73H_ + ΔΔG_K250E_ + ΔΔG_T362I_) or non-additive. Intriguingly, the amino acid substitutions were non-additive for AMP, 2′-dAMP, and GMP incorporation (Figure 4a). In fact, adding together the thermodynamic effects of the four single amino acid substitutions would predict a more catalytically-efficient Sabin enzyme (*i.e.*, ΔΔG is negative) for AMP and GMP incorporation, but less catalytically efficient for 2′-dAMP incorporation (*i.e.*, ΔΔG_D53N_ + ΔΔG_Y73H_ + ΔΔG_K250E_ + ΔΔG_T362I_ > ΔΔG_Sabin_). However, it should be pointed out that these differences are smaller (*i.e.*, |ΔΔG_Sabin_− (ΔΔG_D53N_ + ΔΔG_Y73H_ + ΔΔG_K250E_ + ΔΔG_T362I_)|~0.2–0.3 kcal/mol) than the kT value of 0.6 kcal/mol.

The results with the Sabin I RdRp suggested that there were small allosteric effects among the four amino acid substitutions. Our previous MD simulations with the T362I variant had indicated that the nanosecond timescale dynamics near the region encompassing Asp53 are different from what was observed in the WT enzyme [22]. To gain more insight into potential allosteric effects between these amino acid substitutions, we characterized the double variant D53N/T362I. The sugar selectivity of the double variant was similar to that of the T362I variant, and the nucleobase selectivity was reduced slightly compared to the two single variants D53N and T362I (Table 1). To more rigorously compare the effects of the single-substituted variants to the double-substituted variant, we also determined the thermodynamic effects, as we had for the Sabin variant. The effects of the single amino acid substitutions were non-additive for AMP, 2′-dAMP, and GMP incorporation (*i.e.*, ΔΔG_D53N/T362I_ < ΔΔG_D53N_ + ΔΔG_T362I_), which suggests negative cooperativity between the T362I and D53N substitutions (Figure 4a). It is also interesting to note that the double variant D53N/T362I had similar ΔΔG values for AMP and GMP incorporation as the Sabin variant.

In the case of the triple variant D53N/Y73H/T362I, the effects of the D53N, Y73H, and T362I substitutions appeared to be additive (Figure 4a), which was surprising considering the non-additive results with the D53N/T362I and Sabin variants. To gain more insight, we constructed thermodynamic cycles to gauge the contributions of the Y73H and K250E substitutions to the triple and Sabin variants respectively (Figure 4b–d). Based on these thermodynamic cycles, there were small differences between ΔΔG_D53N/Y73H/T362I_ and the sum of ΔΔG_Y73H_ and ΔΔG_D53N/T362I_, with the largest difference being associated with 2′-dAMP incorporation. There were also small differences between ΔΔG_Sabin_ and the sum of ΔΔG_K250E_ and ΔΔG_D53N/Y73H/T362I_. Here, the largest difference was associated with GMP incorporation.

These results indicated that the Y73H and K250E substitutions provide small adjustments to the catalytic efficiency of the Sabin polymerase, which also has consequences for RdRp fidelity. To better visualize these changes, we compared the *k*_pol,correct_/*k*_pol,incorrect_ values between the T362I, D53N/T362I, D53N/Y73H/T362I, and Sabin variants (Figure 4e,f). It should be kept in mind that this series of variants does not necessarily recapitulate the order that these amino acid substitutions arose during the selection of the Sabin I virus. Nonetheless, this analysis provides some additional insight into a potential selection process in regards to the Sabin I polymerase, keeping in mind that other mutations in the Sabin I virus may have also played a role in regards to the polymerase changes (e.g., K250E is not stable in the Mahoney background). The results indicated that the Y73H substitution induced an increase in the relative rates of nucleotide misincorporation when comparing the D53N/T362I and D53N/Y73H/T362I variants. Interestingly, the Y73H effects appear to be opposed by the K250E substitution when comparing the D53N/Y73H/T362I and Sabin variants, such that the relative rate of the Sabin variant for misincorporation of nucleotide with incorrect nucleobase is very near that of WT RdRp, whereas the relative rate for misincorporation of nucleotide with incorrect sugar is near that of the T362I variant (Table 1, Figure 4).

### 2.5. Structural Dynamic Differences between the Sabin I RdRp and the Triple Variant D53/Y73H/T362I

**Figure 5:**
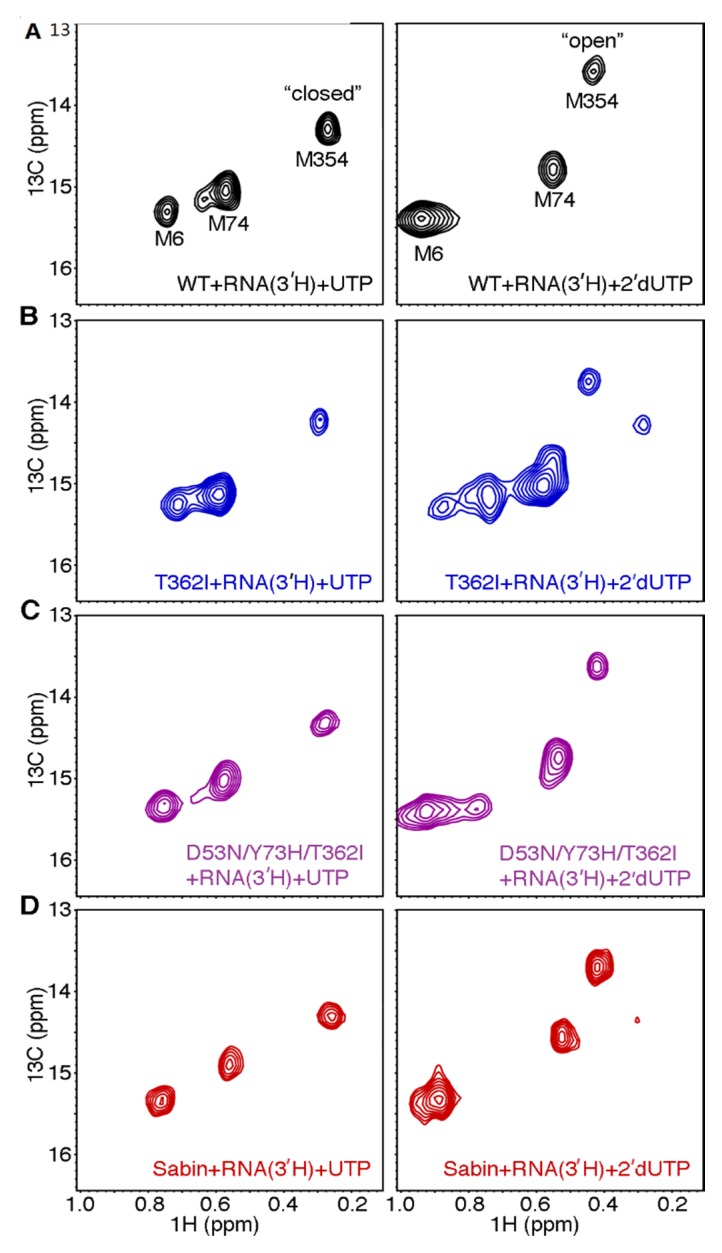
The Sabin I RdRp does not readily populate the closed conformation when incorrect nucleotide binds. [*Methyl-*^13^C] Met ^1^H-^13^C HSQC spectra for (**A**) WT; (**B**) T362I; (**C**) D53N/Y73H/T362I; and (**D**) Sabin I RdRp when the enzyme is bound with s/s RNA lacking a 3′-OH group and (**left**) correct UTP nucleotide or (**right**) incorrect 2’-dUTP nucleotide. Spectra were collected at 293 K with 250 μM RdRp, 1000 μM s/s RNA, and 4 mM UTP or 8 mM 2′-dUTP.

We had previously shown that there is a correlation between RdRp fidelity and the conformational state of motif D, as reported on by the [*methyl-*^13^C]Met ^1^H-^13^C HSQC NMR spectra [22,29,30]. In the NMR experiments, we first add 3′-dATP to s/sU RNA and enzyme, so that the nucleotide will become incorporated but lead to chain termination. Following passage through a de-salting column, another nucleotide is added to form a ternary complex between the enzyme, RNA, and the incoming nucleotide [29]. The correct nucleotide is considered to be UTP as it will basepair to A, which is the next templating base in the RNA. We have suggested that the peak position(s) of Met354 in motif D reports on whether motif D is in an open or closed conformation, which determines whether Lys359 is in a position to act as a general acid [29]. For WT enzyme, the ternary complexes bound with UTP and 2’-dUTP yield different [*methyl-*^13^C] Met NMR spectra, providing fingerprints for the closed and open conformations, respectively (Figure 5a). We have also previously shown that Met354 in the T362I ternary complex bound with 2′-dUTP gives rise to two resonances, consistent with this region of the enzyme fluctuating between the open and closed conformations on the slow NMR timescale (Figure 5b); the Met6 and Met74 peak also provide evidence of conformational exchange. We have suggested that T362I RdRp has reduced nucleotide discrimination because the motif-D loop can more readily access a closed conformation, even in the presence of an incorrect nucleotide [22].

It was very interesting to note that there were differences in both sugar and nucleobase discrimination between the triple variant D53N/Y73H/T362I and the Sabin variant (Table 1; Figure 4). The Sabin I RdRp, in particular, had the lowest sugar discrimination among all variants tested (Table 1). To gain more insight into structural changes that might account for these functional behaviors, we collected [*methyl-*^13^C] Met NMR spectra for the triple variant D53N/Y73H/T362I and Sabin variant. The ternary complexes bound with UTP yielded [*methyl-*^13^C] Met spectra very similar to that of WT enzyme (Figure 5c,d). However, the triple variant D53N/Y73H/T362I and Sabin variant yielded small differences in the NMR spectrum for the ternary complex bound with 2′-dUTP (Figure 5c,d). In these cases, Met6 gave rise to two (or more) peaks, suggesting conformational exchange around this residue. We note that the ε-methyl group of Met6 packs against Phe59, which is located on the small α-helix that is N-capped by Asp53. Perhaps most informative is the difference between the Met354 peak(s) in the triple and Sabin variants compared to the T362I variant. There was no evidence for two Met354 resonances in the D53N/Y73H/T362I ternary complex bound with 2′-dUTP, suggesting that the enzyme was not substantially populating the closed conformation. For the Sabin variant, there was a very low intensity peak at the chemical shift position we expect for Met354 in the closed conformation. These results suggest that the Sabin 2’-dUTP ternary complex populates the closed conformation to a greater extent than the corresponding complexes for WT RdRp and the triple variant D53N/Y73H/T362I, but not nearly to the degree populated by the T362I variant.

### 2.6. The Sabin I RdRp is More Susceptible to 2′-Modified Nucleotides

It was interesting to note that the Sabin I RdRp more readily incorporates 2′-dAMP than WT RdRp, especially in light of the 2′ modified nucleotide derivatives that have been very successful in treating RNA virus infections, especially Hepatitis C virus [44,45]. As such, we characterized the ability of WT and Sabin RdRp to incorporate 2′-C-methylAMP (Table 1). Intriguingly, comparisons of the second-order rate constants k_pol_/K_d,app_ indicated that Sabin RdRp more readily incorporated 2′-C-methylAMP by a factor of two compared to WT RdRp. This result may suggest that Sabin I RdRp is more susceptible to incorporating 2′-modified nucleotides than WT enzyme.

## 3. Discussion

The Sabin live, attenuated vaccine has been a major component of the global efforts to eradicate poliovirus. It has been previously suggested that changes in the IRES are the major factor contributing to the attenuation of the virus [13,14]. Nonetheless, other mutations in the vaccine strains may also contribute to viral attenuation and efficacy of the vaccine [15,16,17,18,19,20,22]. We were especially interested in the RdRp amino acid changes encoded by the Sabin I strain. Our previous studies had shown that the T362I amino acid substitution induces a lower fidelity polymerase, and viruses encoding the T362I change showed a statistically significant decrease in viral pathogenesis [22]. We were thus interested in determining if there are similar functional changes in the Sabin I RdRp carrying all four amino acid changes (*i.e.*, D53N/Y73H/K250E/T362I). To our surprise, the Sabin I RdRp discriminates against nucleotides with incorrect nucleobases to the same extent as WT enzyme (Table 1; Figure 3), suggesting that the other three amino acid changes (D53N, Y73H and K250E) also modify RdRp function.

We also attempted to initiate cell-based assays with recombinant PV (*i.e.*, Mahoney background) encoding the Sabin I RdRp, but the K250E change was not genetically stable and virus recovered from HeLa cells only retained the D53N/Y73H/T362I changes. It should be kept in mind that the PV RdRp (also known as 3D) is found within other polyproteins, including 3CD, and changes in the 3D domain may have effects on the functions of these other proteins. In fact, 3CD is known to interact with the 5′ untranslated region (UTR) [46,47,48] and these interactions are important for regulating RNA synthesis and protein translation [48,49]. A change from a positively-charged residue (*i.e.*, Lys) to a negatively-charged residue (*i.e.*, Glu) at position 250 may disrupt interactions between 3CD and the 5′UTR. The loss of the K250E change in the Mahoney background may suggest that other elements in the Sabin I virus are important for retaining this mutation, or from a different perspective, the K250E change may have been necessary to compensate for other mutations in the Sabin I strain during the selection process, such as those in the 5′UTR.

The lack of selective pressure to maintain WT-levels of sugar discrimination may have allowed the Sabin I RdRp to drift towards a lower sugar selectivity, even lower than that of the T362I variant (Table 1; Figure 3). We had previously suggested that the lower fidelity of the T362I variant is likely because this substitution allows the motif-D active-site loop to fluctuate more readily into a “closed” conformation even in the presence of incorrect nucleotide [22]. However, this reasoning likely does not fully explain the lower sugar selectivity of the Sabin I RdRp. The equilibrium population of the “closed” state, according to the NMR experiments (Figure 5), is lower for the Sabin I RdRp compared to the T362I variant. It should be kept in mind, however, that these NMR experiments are under equilibrium conditions, and so the open-closed transition may still occur more rapidly in the Sabin I enzyme compared to WT RdRp, but not be reflected in the NMR spectra. Nonetheless, the NMR experiments suggest that the lower sugar fidelity of the Sabin I RdRp may owe to factors outside those of the motif-D active-site loop. The lower sugar selectivity of the Sabin I RdRp and RdRps associated with VAPP may serve as their Achilles heel. We have also shown that Sabin I RdRp more readily incorporated nucleotides with modified sugar groups (Table 1). These results suggest that Sabin I may be more sensitive to 2’-modified nucleotide analogs, which have been used to treat Hepatitis C and related viruses [44,45]. Considering that all variants we tested have lower sugar fidelity than WT RdRp, VAPP may also be more sensitive to this class of compounds, potentially providing an additional treatment option.

We have previously suggested that the small change in fidelity for the T362I variant likely contributes to viral attenuation [22]. In contrast, our results here suggest that fidelity of the Sabin I RdRp likely would not contribute to viral attenuation. These results would be consistent with previous studies that suggest that mutations in the IRES primarily contribute to viral attenuation in the Sabin strain [13,14]. Nonetheless, other RdRp functions might be impacted by the Sabin substitutions. It is already known that the Y73H change interferes with RdRp initiation [19]. The Sabin changes may also affect interactions with other viral or host proteins, including when the RdRp is found as a domain in other important viral polyproteins (e.g., 3CD). In these cases, the Sabin I substitutions may also be acting cooperatively to fine-tune these other functions.

## 4. Materials and Methods

### 4.1. Materials

[γ-^32^P]ATP and [α-^32^P]UTP (>7000Ci/mmol) was from VWR-MP Biomedical (Santa Ana, CA, USA); nucleoside 5′-triphosphates and 2′-deoxynucleoside 5′-triphosphates (all nucleotides were ultrapure solutions) were from GE Healthcare Bio-Sciences (Pittsburgh, PA, USA). 3′-Deoxyadenosine 5′-triphosphate (cordycepin) was from Trilink Biotechnologies (San Diego, CA, USA). All RNA oligonucleotides were from Dharmacon Research, Inc. (Boulder, CO, USA). T4 polynucleotide kinase was from New England Biolabs, Inc (Ipswich, MA, USA). [*Methyl*-^13^C] methionine was from Cambridge Isotope Laboratories (Tewksbury, MA, USA). HisPur Ni-NTA resin was from ThermoFisher Scientific (Waltham, MA, USA). Q-Sepharose fast flow resin was from GE Healthcare Bio-Sciences. Polyethylenimine-cellulose thin layer chromatography (TLC) plates were from EM Science (Gibbstown, NJ, USA). QuickChange site-directed mutagenesis kit was from Stratagene (La Jolla, CA, USA). The plasmid DNA isolation miniprep kit was from Qiagen (Frederick, MD, USA). All other reagents were of the highest grade available from Sigma-Aldrich (St. Louis, MO, USA) or ThermoFisher.

### 4.2. Plasmid Construction

All RdRp variants including D53N, Y73H, K250E, D53N/T362I, D53N/Y73H/T362I, and D53N/Y73H/K250E/T362I (Sabin variant) were generated using the QuickChange site-directed mutagenesis kit using appropriate forward and reverse primers. Mutations were confirmed by DNA sequencing at the Nucleic Acid Facility at the Pennsylvania State University. It should be noted that all RdRp constructs contain two additional interface I amino acid substitutions (L446D and L455D) to reduce protein oligomerization.

### 4.3. Overexpression and Protein Purification

The overexpression and protein purification were conducted by following the procedure previously described [30,50,51,52].

### 4.4. Kinetic Assays

The kinetic assays including the active site titration assay, assembly assay and the dissociation assay were conducted as described previously [30,51]. Briefly, the reaction contained 50 mM HEPES pH 7.5, 10 mM 2-mercaptoethanol, 5 mM MgCl_2_, and 60 μM ZnCl_2_. The stopped flow experiments and the benchtop assays for nucleotide incorporation were conducted as described previously [22,29,34]. Reactions were incubated at 30 °C and quenched by the addition of an equal volume of ethylenediaminetetraacetic acid (EDTA) to a final concentration of 25 mM. Specific concentrations of RdRp, s/sU RNA, and nucleotide are indicated in the corresponding figure legend.

### 4.5. Determination of Kinetic Constants (K_d,app_, and k_pol_) for Nucleotide Incorporation Catalyzed by RdRp

Kinetic data analysis was conducted by following the procedure and equations previously described [22,29]. Briefly, the quenched product samples from kinetic assays were analyzed by 23% highly cross-linked denaturing polyacrylamide gel. The gel was visualized using a PhosphorImager and quantified using the ImageQuant software (GE Healthcare Bio-Sciences). The data were fit into different curves using Kaleidagraph (Synergy Software, Reading, PA, USA).

### 4.6. NMR Sample Preparation and Spectroscopy

NMR sample preparation followed procedures described previously using [*methyl*-^13^C] Met-labeled PV RdRp [29,30]. ^1^H, ^13^C HSQC (heteronuclear single quantum coherence) NMR spectra were collected on a Bruker Avance III 600 MHz spectrometer equipped with a 5-mm “inverse detection” triple-resonance (^1^H, ^13^C, ^15^N) single axis gradient TCI probe at 293 K. NMR samples generally contained 250 μM RdRp, 1000 μM s/s RNA, and 4 mM UTP, 8 mM 2′-dUTP or 16 mM ATP.

### 4.7. Construction of Mutated Viral cDNA Clones and Replicons

To introduce the four mutations, D53N, Y73H, K250E and T362I, into the RdRp/3D^pol^-coding sequence of viral cDNA, pMovRA, overlap PCR was performed with oligonucleotides PV-3D-D53N-for (5′-GAT CCC AGG CTT AAG ACA AAT TTT GAG GAG GCA ATT TTC-3′), PV-3D-D53N-rev (5′-AGA AAA TTG CCT CCT CAA AAT TTG TCT TAA GCC TGG GAT C-3′), PV-3D-Y73H-for (5′-ATT ACT GAA GTG GAT GAG CAT ATG AAA GAG GCA GTA GAC-3′), PV-3D-Y73H-rev (5′-GTC TAC TGC CTC TTT CAT ATG CTC ATC CAC TTC AGT AAT-3′), PV-3D-K250E-for (5′-GCT TGG TTC GAG GCA CTA CAA ATG GTG CTT GAG AAA ATC GGA-3′), PV-3D-K250E-rev (5′-TCC GAT TTT CTC AAG CAC CAT TTC TAG TGC CTC GAA CCA AGC-3′), PV-3D-*Bgl*II-for (5′- GGC AAA GAA GTG GAG ATC TTG GAT GCC AAA GC-3′), PV-3D-*EcoR*I-*Apa*I-polyA-rev (5′-CGC TCA TCG ATG AAT TCG GGC CCT TTT TTT TTT TTT TTT TTT TCT CC-3′) and the pMoV-3D-BPKN-I92T-T362I plasmid as template [22]*.* PCR products were purified and digested with *Bgl* II and *Eco* RI and the digested PCR product was ligated into pMovRA vector.

### 4.8. RNA Transcription

The pMo-3D-D53N-Y73H-K250E-T362I plasmid was linearized with *Apa*I and purified with Qiaex II suspension (Qiagen) by following the manufacturer’s protocol. RNA was then transcribed from the linearized plasmid DNAs in a 20-μL reaction mixture containing 350 mM HEPES, pH 7.5, 32 mM magnesium acetate, 40 mM dithiothreitol (DTT), 2 mM spermidine, 28 mM nucleoside triphosphates, 0.025 μg/μL linearized DNA, and 0.025 μg/μL T7 RNA polymerase. The reaction mixture was incubated for 4 h at 37 °C, and magnesium pyrophosphate was removed by centrifugation for 2 min. The supernatant was transferred to a new tube, and RQ1 DNase (Promega; Madison, WI, USA) was used to remove the template. The RNA concentration was determined by measuring absorbance at 260 nm, assuming that an *A*_260_ of one was equivalent to 40 μg/mL, and the RNA quality was verified by 0.8% agarose gel electrophoresis.

### 4.9. Infectious Center Assays

HeLa cells were transfected by electroporation with 5 µg of viral RNA transcript and these cells were serially diluted and plated onto HeLa cell monolayers. Cells were allowed to adhere to the plate for 1 h at 37 °C and then the medium/PBS was aspirated. Cells were covered with 1X DMEM/F12 plus 10% fetal bovine serum and 1% agarose. After 2–4 days of incubation, the agarose overlay was removed and the cells were stained with crystal violet.

### 4.10. Virus Isolation, RNA Isolation, cDNA Synthesis, and Sequencing to Confirm the Presence of the Quadruple Mutation

HeLa cells were transfected by electroporation with 5 µg viral RNA transcript, added to HeLa cell monolayers and incubated at 37 °C. Upon cytopathic effect (CPE), viruses were harvested by three repeated freeze-thaw cycles, cell debris removed by centrifugation. Viral RNA was isolated with QiaAmp viral RNA purification kit (Qiagen), as recommended by the manufacturer. The 3Dpol cDNA was prepared from purified viral RNA by reverse transcription with MMuLV-RT (New England Biolabs) with Random Hexamer Primers (manufacturer). The resulting DNA product was then PCR amplified using SuperTaq DNA polymerase (Ambion; Naugtuck, CT, USA) and oligonucleotides PV-3D-*Bgl*II-for and PV-3D-*EcoR*I-*Apa*I-polyA-rev as primers. The presence of all four mutations was determined by sequencing of the nucleic acid obtained in second PCR step with oligonucleotides PV-3D-seq100-for (5′-GAA GGG GTG AAG GAA CCA G-3′) and PV-3D-seq500-for (5’-AGG TTG AGC AGG GGA AA-3′).
